# Laser–Arc Hybrid Cladding of Al-Mg Alloy Coating on AZ80 Mg Alloy: Effect of Laser Beam Oscillations Amplitude

**DOI:** 10.3390/ma15207272

**Published:** 2022-10-18

**Authors:** Zhiqiang Ren, Yang Zhao, Guofeng Han, Wenyu Wang, Kebin Zhou, Tianpeng He, Yu Sun

**Affiliations:** National Key Laboratory for Remanufacturing, Army Academy of Armored Forces, Beijing 100072, China

**Keywords:** laser–arc hybrid welding, magnesium alloy, cladding, wear resistance, hardness

## Abstract

The effect of beam oscillating amplitude on the microstructure and performance of AZ80 Mg alloy cladded with Al-Mg alloy coating by laser–arc hybrid welding was studied. The penetration depth decreases significantly while welds are widened because of the increase in the oscillating area of a laser beam. Alloy segregation and keyhole-induced porosity can be suppressed by the laser beam oscillation. With the increase in the oscillating amplitude, the Al distribution becomes uniform in the weld seam because of the rapid and fierce stirring by the oscillating laser. However, the diluting of the cladded Al alloy restrains the formation of the brittle Mg_17_Al_12_ phase, and then causes the weakening of hardness and wear resistance of the cladded layer. Considered comprehensively, the optimized oscillating amplitude was 1 mm, which can produce the weld seam with good appearance, fewer segregation and porosity defects, and acceptable hardness and wear resistance.

## 1. Introduction

Mg alloys are considered to have the highest strength-to-weight ratio of structural alloys. Besides their low density and high specific strength, Mg alloys possess excellent damping capacity, good cast-ability, machinability, and recyclability [1,2,3]. Thus, Mg alloys are especially valuable to the automotive and aerospace industries because of the benefits to energy conservation and higher fuel efficiency through weight reduction [4,5,6]. Unfortunately, the low hardness, and poor wear and corrosion resistance of Mg alloys hindered their wide application in the engineering fields, especially in a severe friction, wear, and corrosive environment [7,8].

To overcome these drawbacks, cladding and alloying techniques are employed to enhance the surface properties of Mg alloy [9,10,11]. Al alloys were usually employed as protection layers on the surface of Mg-alloy components, since Al exhibits a good metallurgy consistency with the Mg and greatly improves the strength and corrosion resistance of Mg alloys [12,13,14]. A variety of melting technologies have been developed to deposit alloy layers on components for cladding [15], dissimilar joints [16], and additive manufacturing [17], such as selective laser melting [18,19,20], cold-arc melting [21,22], electron-beam melting [23,24,25], and so on. These high-energy melting methods have become advanced technologies in the surface engineering and remanufacturing engineering of Mg alloy components, since rapid heating, melting, and cooling provide extreme non-equilibrium conditions that cannot be achieved by conventional methods, so that the parts formed gain excellent comprehensive properties [26,27,28].

Recently, laser–arc hybrid welding (LAHW) technology has been receiving considerable attention in Mg-alloy cladding because the combination of laser and arc heat sources can offer many advantages [29,30]. In the hybrid laser-arc welding process, the laser beam and the electric arc interact in a common weld pool, and the hybridization effect leads to deeper penetration, higher welding speeds, wider gap tolerance, better weld bead surface appearance, and reduced welding defects, leading to a smaller porosity, which makes it possible to increase productivity and improve weld quality simultaneously [31,32,33,34]. LAHW is complex and includes numerous processing parameters and challenges. Porosity is the most frequently occurring defect in laser–arc hybrid welding [35,36]. When the solidification rate of molten metal is higher than the back-filling speed of molten metal, the porosity is formed because of improper welding parameters. Porosity formation and mitigation in LAHW is more complicated since two sources are introduced in welding [37]. A great amount of research has been done to balance welding parameters for porosity suppression—for example, laser and arc power [38], arc position [39], torch angle [40], and welding speed [41]. Besides the optimizing of welding parameters, some novel methods have been developed to mitigate porosity, such as laser beam oscillations [42,43], magnetic fields [44,45], and ultrasonic vibration [46,47]. Among these novel methods, laser beam oscillating welding is an attractive method because it can effectively enhance the mechanical properties by weakening macro-segregation, inhibiting welding cracks, and mitigating porosity [48]. Wang et al. [43] use circular beam oscillations for LAHW of 8 mm AA6082, where the porosity was reduced from 5% to <1%. Hu et al. [42] found that the mechanical properties of a welded joint could be significantly improved via beam oscillation welding. The maximum tensile strength of an AA6061-T6-welded joint reaches 277 MPa (about 89% of the AA6061-T6 sheets) and the elongation improves by about 65% compared to the sample with non-oscillation welding. Zhang et al. [49] stated that the lower porosity induced by an oscillating laser beam improved the tensile strength of the Al-6Mg alloy lock butt weld, which was 36.3% higher than that of the weld without oscillation. Wang et al. [50] studied three oscillating paths—linear, circular, and infinity—on the laser beam oscillating welding of 5A06 aluminum alloy sheets. Among the three oscillating paths, the infinity mode was the best in terms of decreasing the porosity in the weld and increasing the tensile strength.

In this paper, AZ80 Mg-alloy plates were cladded with 5356 Al alloy by LAHW. The effects of the oscillating amplitude on the weld geometry, internal defects, and microstructure were investigated. The relationship between the oscillating amplitude and wear resistance of the cladding layer were discussed.

## 2. Experimental Details

### 2.1. Raw Materials and Equipment

AZ80 alloy plates with dimensions of 200 × 100 × 10 mm were used as the substrate. 5356 Al-alloy filler metal with a 1.2 mm diameter was used for welding. The actual chemical composition of the substrate metals and welding wire are presented in Table 1.

**Figure 1:**
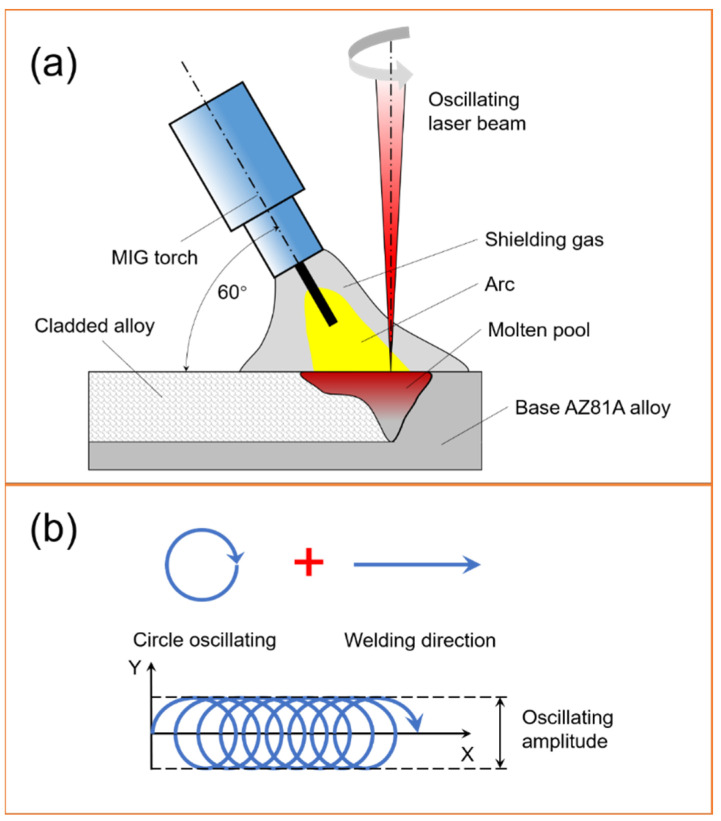
Schematic of (**a**) LMHW set-up and (**b**) laser beam oscillation pattern.

The cladding experiments were carried out by a LMHW system composed of an IPG YLR-6000 fiber laser (Beijing, China), a laser head, a FRONIUS TPS4000 MIG arc welder (Wels, Austria), and a FUNAC M-710ic/50 robot (Yamanashi, Japan). The LMHW set-up and laser beam oscillation pattern are shown in Figure 1. The angle between the arc torch centerline and the workpiece surface was 60°. The distance between laser and arc was 2 mm and the wire extension was 15 mm. Table 2 shows the parameters of the LAHW process and the whole experiment process is depicted in Figure 1. Before welding, the AZ80 alloy plates were polished with 600# (standard ANSI grit) SiC abrasive paper in a cabin filling with argon and cleaned with alcohol to remove the oxidation layer. After laser cladding, the surface of the cladded layer was grinded with a steel brush, and then cleaned by alcohol to remove the oxide. The experiments were performed with varied beam-oscillating amplitudes (0, 0.5, 1.0, 1.5, and 2.0 mm), and the corresponding samples were designated as A0, A0.5, A1.0, A1.5, and A2.0, respectively.

### 2.2. Phase Composition and Microstructure of Cladded Layer

The welded samples were sectioned and polished for metallurgical examination using standard metallographic preparation techniques. Samples were ground with SiC paper with grit sizes varying from 400 to 1500. After grinding, the samples were thoroughly washed with water and subsequently polished with 9, 3, and 1 μm diamond paste suspension. Etching was performed with 2 mL HCl, 6 mL HNO_3_, and 92 mL of distilled water.

Optical micrographs for each welding condition were captured and analyzed using an Olympus GX51 optical microscope. Microstructure observations from sections of the interlayer and top layer were investigated by a FEI Quanta 200 scanning electron microscope (SEM, Eindhoven, The Netherlands) with an energy-dispersive X-ray spectroscopy (EDS). The phase compositions of the interlayer and top layer were analyzed by Philips Xpert-Pro (Amsterdam, The Netherlands) type X-ray diffraction (XRD) with Cu Kα radiation, scanning angles of 20°~80°, and a scanning speed of 5°/min.

### 2.3. Properties of Cladded Layer

The microhardness was measured from a cross-section of the sample using an HVS-1000 microhardness tester with a load of 300 g and loading time of 15 s. The coefficient of friction and wear properties were evaluated using an HT-1000 wear tester with a normal load 5 N, a time of 30 min, and a frequency of 5 Hz at room temperature. GCr15 balls of 4 mm were used as the friction pair.

## 3. Results and Discussion

**Figure 2:**
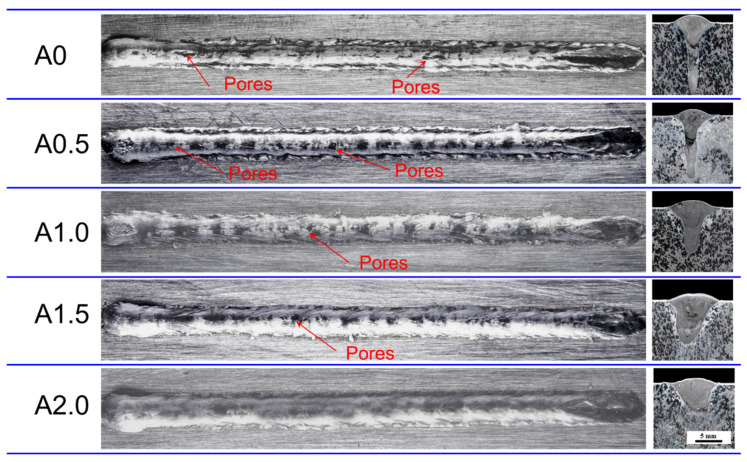
Typical macroscopic morphologies of the weld seam appearance and profile of 5356 Al alloy cladded on AZ80A by LAHW with different oscillating amplitudes.

Figure 2 exhibits the typical macroscopic appearances of the weld seam appearance and cross-section profile with different laser oscillating amplitudes. As seen in Figure 2, the macroscopic appearance of the weld seam without oscillating amplitude (A0) is rough and plenty of porosities, whose diameters are mostly over 1 mm, are in the seam centerline. With the increase in oscillating amplitude, the macroscopic appearance of the coating exhibits it as much smoother and the number of pores has decreased significantly, meaning that appearance quality of the coating can be improved by employing oscillating amplitude. As for the cross-section profile, the seam changed from V-shape to U-shape with the increase in oscillating amplitude, which was reported in the previous literature [42]. Generally, the molten pool and weld seam of LMHW was divided into two zones. In the upper zone, the long and shallow part of the molten pool is the arc zone, which is formed by the action of the arc and keyhole. In the lower part is the laser zone, which results from the action of the laser keyhole [43]. In particular, the laser keyhole phenomenon disappeared when A ≥ 1.0 mm, which agrees with the previous studies. The macro profile presents a dark grey color in the arc zone, while exhibiting a light grey in the laser zone. When A ≥ 1.0 mm, the samples exhibit the similar color in the whole weld seam profile. It is worth noting that a diffusion area between the arc zone and laser zone especially in sample A0 and A0.5. The dark and grey color were simultaneously existing in this diffusion area. The different color indicates the different phase in the weld seam profile.

**Figure 3:**
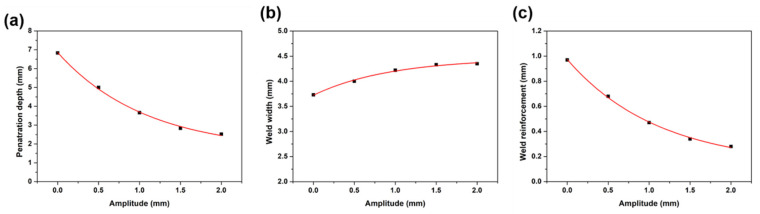
(**a**) penetration depth, (**b**) weld width, (**c**) weld reinforcement.

To further analyze the seam profile, the variation of penetration depth, weld width, and weld reinforcement were presented in Figure 3. It was obvious that the penetration depth and weld reinforcement were decreased with the increase in oscillating amplitude, while the weld width increased. The broadening of the weld width mainly results from the laser beam moving to a larger area and transferring the heat to a bigger area [42]. The decrease in penetration depth mainly results from the fast movement of the laser beam, which restrains the focusing of the laser energy and the laser keyhole effect [49]. When A ≥ 1.0 mm, the weld penetration depth is less than 4 mm and the laser action zone disappears, which means the welding mode is conduction mode. When A ≤ 0.5 mm, the weld penetration depth is over 5 mm, and the welding mode is keyhole mode. It is worth noting that the welding mode transfers from keyhole mode to conduction mode when the amplitude is larger than 1.0.

**Figure 4:**
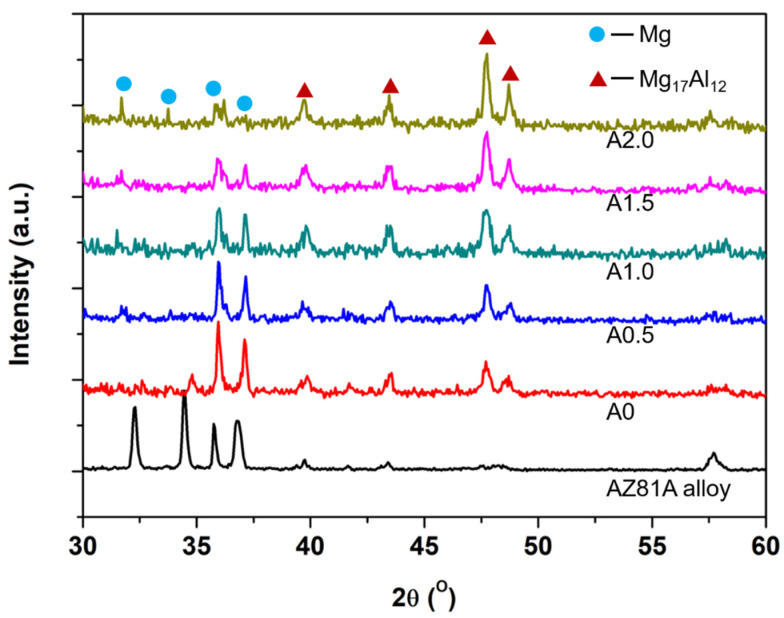
XRD patterns of the based AZ81A alloy and weld seam profiles cladded with different oscillating amplitude.

The composition of the weld seam was detected by XRD, which is shown in Figure 4. The weld seam is mainly composed of α-Mg and Mg_17_Al_12_ phases, which can be proved by PDF 01-089-4244 and PDF 01-073-1148, respectively. It is noted that the content of the α-Mg phases in the weld seam decreases with the increase in oscillating amplitude, while the Mg_17_Al_12_ phases increase with the increase in oscillating amplitude. It is worth noting that the Al phase was not found in these five samples, which suggests that melted Al was mixed with melted Mg alloy and formed the Mg_17_Al_12_ phases.

**Figure 5:**
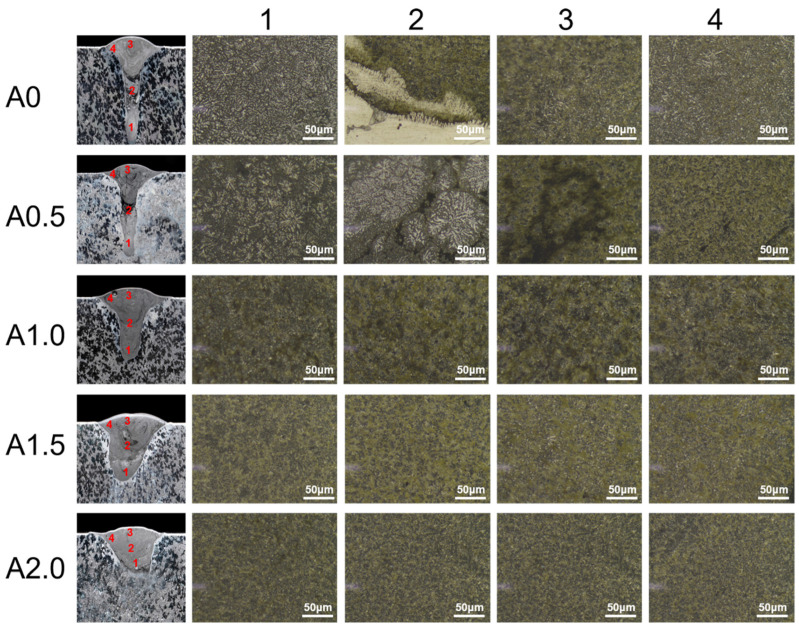
Optical microscope images of the cross-section profile of the weld seam cladded with different oscillating amplitudes.

Figure 5 presents the microstructures of the welded seam profiles cladded with different oscillating amplitudes. The oscillating amplitude shows significant impact on the microstructures. When the oscillating amplitude is zero and 0.5, the bottom of the welded seam presents a dendritic structure. In contrast, the bottom of the welded seam presents a fine equiaxed grain structure when A ≥ 1.0 mm. According to the XRD results, it can be deduced that the dark area was α-Mg and the bright area was the Mg_17_Al_12_ phases. The precipitated Mg_17_Al_12_ phase with a dendritic structure results from the solidification of the laser-melted Mg-Al alloy. With the stirring effect of the laser beam oscillations, the dendritic structure Mg_17_Al_12_ phase was destroyed to form a fine equiaxed grain structure. Moreover, the stirring effect of the laser beam oscillations promotes the diffusion of the Al element, leads to the increase in Al content in the bottom area, and causes the increase in the bright Mg_17_Al_12_ phase. In the position of point 2, the samples A1.0, A1.5, and A2.0 present a similar equiaxed grain structure to that in point 1. However, the microstructure of the A0 and A0.5 samples in position 2 exhibits two distinct phases: one is in grey and another is in white. These two phases were segregated in the A0 sample but mixed in the A0.5 sample. This phenomenon mainly results from element diffusion between the Mg melted by laser and the Al melted by arc, which forms a turbulence area between the arc zone and laser zone [49]. In the point 3 and 4 areas, some bright Mg_17_Al_12_ phases were present in the stick structure in the A0 sample. The segregation of the dark α-Mg phase can be found in the A0.5 and A1.0 samples. In contrast, the A1.5 and A2.0 samples present a fine eutectic mixture structure. The stirring effect of the laser beam oscillations improves the melt flow and leads to the forming of a fine eutectic structure in the weld seam [50].

**Figure 6:**
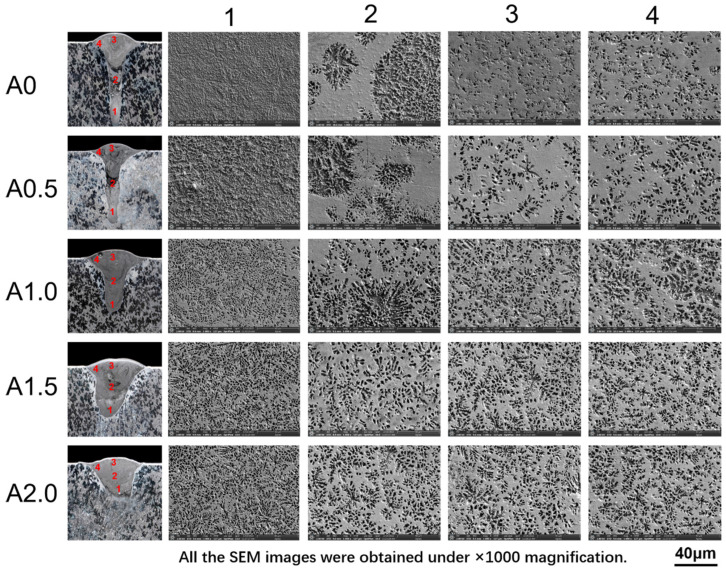
SEM image of the weld seam cladded with different oscillating amplitudes.

**Figure 7:**
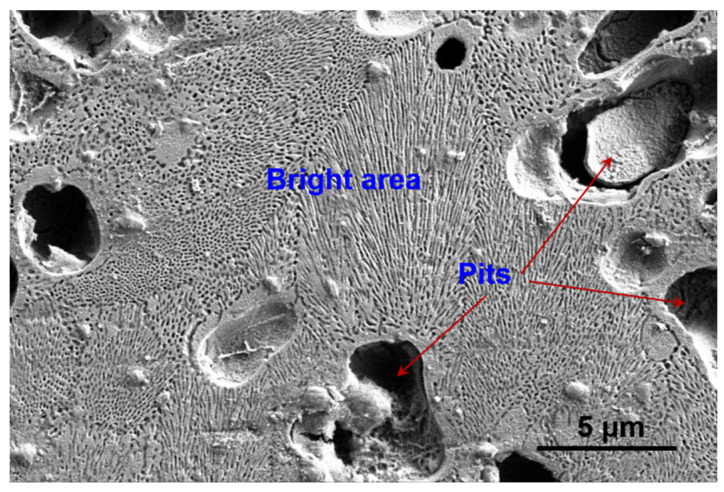
Typical microstructure of the seam cross-section.

The cross-section of the weld seam was observed by SEM in different positions (Figure 6), which were pointed in the left picture. According to the XRD results, the weld seam is mainly composed of α-Mg and Mg_17_Al_12_. The bright area was mainly the Mg_17_Al_12_ phase because it was difficult to etch, while the dark area was mainly the α-Mg phase, as the α-Mg was easy to erode to form a corrosion pit. Figure 7 shows the typical microstructure of the bright area and dark area in the SEM images. These pits with diameters larger than 2 μm result from the erosion of α-Mg. The bright area was like a honeycomb structure, composed of nanoscale pits and frames. These nanoscale pits were formed by the erosion of the secondary precipitated α-Mg phase. The frame was the Mg_17_Al_12_ phase precipitated from the weld seam.

**Figure 8:**
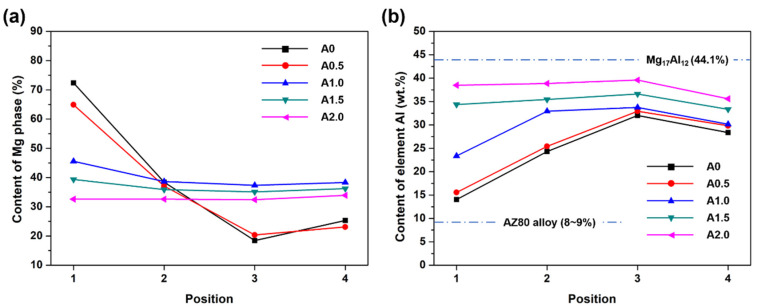
α-Mg phase content (**a**) and Al element content (**b**) in different positions of the weld seam cladded with different oscillating amplitudes.

To further analyze the content of Al and Mg in different areas, these areas were analyzed by EDS. Obviously, position 1 in both the A0 and A0.5 samples is mainly a matrix AZ80 alloy. The Al mass content is about 13~15%, which is near the content in matrix AZ80 alloy (Figure 8b). When A ≤ 0.5 mm, the influence of laser oscillating was weak; the laser keyhole effect causes the melt of the matrix AZ80 alloy. When A ≥ 1.0 mm, the effect of laser oscillating was enhanced. Besides the decrease in the penetration depth, the diffusion of Al elements is enhanced with the increase in oscillating amplitude. The content of Al in the bottom area (position 1) of the weld seam dramatically increases (Figure 8b), while the content of the Mg phase decreases and Mg_17_Al_12_ increases (Figure 8a) in samples A1.0, A1.5, and A2.0. In position 2, the Al content increases sharply and the α-Mg phase content decreases in the A0, A0.5, and A1.0 samples. In contrast, the variations of the Al content and α-Mg phase were weak in the A1.5 and A2.0 samples. In position 3, the Al content reaches the highest value for all samples. Among these five samples, the A0 sample possesses the highest Al content in position 3. It is suggested that the A0 sample possesses the greatest Al elements gradient in the weld seam in the depth direction. Attributed to the dilute of the matrix Mg alloy, the Al content in position 4 is slightly decreased compared with in position 3. The Al and Mg elements distribution in various samples further proved that the stirring effect of the laser beam oscillations improves the melt flow and leads to a homogenization of the Al and Mg elements [42].

**Figure 9:**
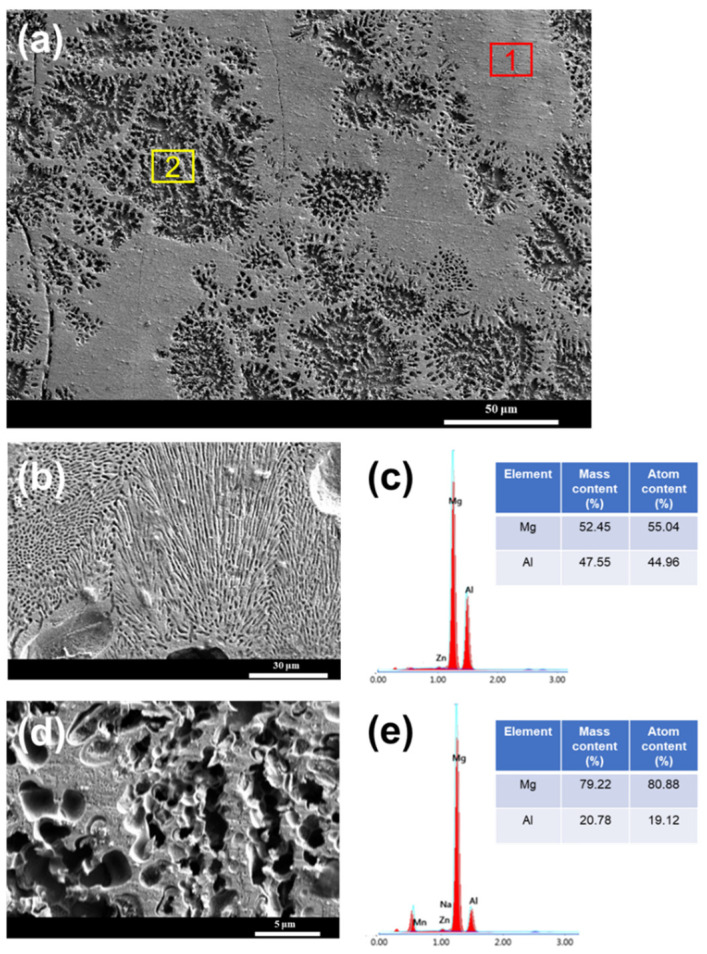
Distribution of Mg and Al elements in the turbulence area between the arc zone and laser zone. (**a**) SEM image of the turbulence area, (**b**) morphology of area 2 in image a, (**c**) EDS results of area 1, (**d**) morphology of area 1 in image a, (**e**) EDS results of area 2.

Obviously, there is a turbulence area between the arc zone and laser zone. To further analyze this area, high-revolution SEM and EDS was employed, as shown in Figure 9. In this area, the bright area might be the Mg_17_Al_12_ phase, while the dark area is the α-Mg phase. The EDS results proved this deduction, where the Mg/Al molar ratio (1.22) near the Mg_17_Al_12_ phase in area 1 and the Mg content (79.22%) is near the matrix alloy (~90%). In the bright area, the Mg_17_Al_12_ phase presents the lamellar structure, which was the typical Mg_17_Al_12_ growth mode. The gaps between the Mg_17_Al_12_ lamellar result from the etching of the secondary-precipitated Mg. In the dendrite area, the α-Mg phase was etched and leaves holes in area 2. The residual was a Mg_17_Al_12_ phase with a dendritic structure.

**Figure 10:**
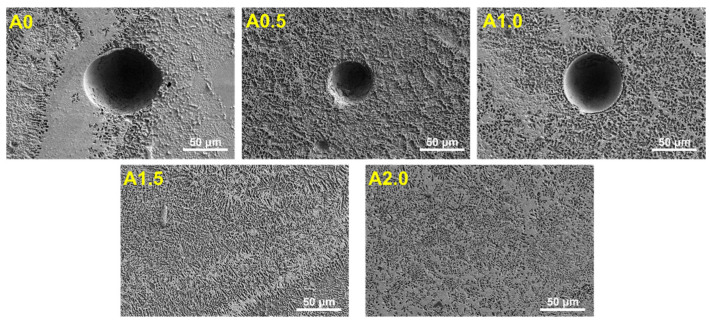
Typical morphology of the in the turbulence area of the weld seam cladded with different oscillating amplitudes.

Except for the segregation of the Mg alloy, some pores can be found in the turbulence area, as shown in Figure 10. These pores appear in a circular shape formed from bubbles. The formation of these pores was attributed to the keyhole-induced porosities. When the vapor jet induced by an incident laser acts on the middle part of the keyhole, it may make the rear wall of the keyhole bulge locally to form a gas cavity [39]. As the keyhole moves forward, the gas cavity changes into a bubble, which will enter the molten pool and flow to the rear with the melt flow. Because the laser zone is narrow, the bubbles can be easily caught by the solidification front before floating up. Finally, the bubbles are trapped in the weld to form circular porosity [40,41]. Thus, pores with about ~200 μm can be easily found in the turbulence area of the A0 sample. With the increase in oscillating amplitude, the size of the pores decreases rapidly. The size of pores decreases to about 100 μm in the turbulence area of the A0.5 sample, while that in the A1.0 sample decreases to about 30 μm. Notably, no pores can be found in the turbulence areas of the A1.5 and A2.0 samples. It was suggested that keyhole-induced porosity can be suppressed by the laser beam oscillation because of the stirring effect of the laser beam oscillating.

### Performance of the Cladded Layer

**Figure 11:**
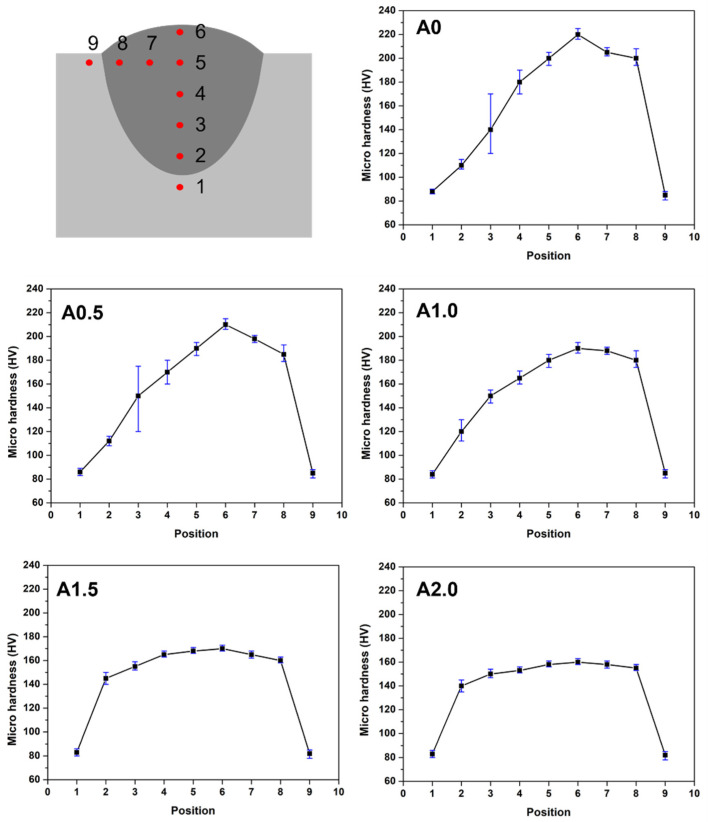
Microhardness of the weld seam cladded with different oscillating amplitudes.

The microhardness of the cladded layer is shown in Figure 10. Nine different points were selected to analyze the microhardness of the cladded layer, as shown in Figure 11a. The points 1, 2, 3, 4, 5, and 6 were in the depth direction of the weld, while 6, 7, 8, and 9 were in the width direction in the top area of the weld. Obviously, the microhardness of the cladded layer exhibits various regulations with different oscillating amplitudes. The microhardness of points 1 and 9 were about 80 HV for all five samples, which was consistent with the value of the matrix alloy. As for point 2, the microhardness of the A0 (~110 HV) and A0.5 (~110 HV) samples was much lower than that of samples A1.5 (~140 HV), and A2.0 (~140 HV). The microhardness of the cladded layer exhibits a great gradient in samples A0, A0.5, and A1.0. The microhardness in the depth direction increases from 80 HV to 220 HV for the A0 sample, while the A0.5 and A1.0 samples increase to 210 HV and 190HV, respectively. The A0, A0.5, and A1.0 samples presented a great Al content gradient that leads to a great gradient microhardness in the depth direction. The microhardness of the cladded layer exhibits a gentle increase in the depth direction for samples A1.5 and A2.0, which were only 170 HV and 160 HV, respectively, for the maximum microhardness. Comparing the maximum hardness of these samples, the variation of the hardness was consistent with the Al content in the top area of the reinforcement. According to microstructure analysis, an increase in Al content leads to the formation of the more brittle Mg_17_Al_12_ phase. The brittle Mg_17_Al_12_ phase results in the enhancing of hardness. In the width direction (points 6, 7, and 8), point 6 presents the highest microhardness. With the point closing to the boundary of the weld seam, the microhardness exhibits a slight decrease because of the dilute of the matrix Mg alloy. Furthermore, the microhardness deviation of points 3 and 4 in samples A0 and A0.5 was abnormal, being much larger than that in samples A1.5 and A2.0. This phenomenon is the segregation of Al-Mg alloy in the turbulence area. The Al-element-abundant area possesses the more brittle Mg_17_Al_12_ phase, exhibiting high microhardness, while the Mg-element-abundant area presents low microhardness.

**Figure 12:**
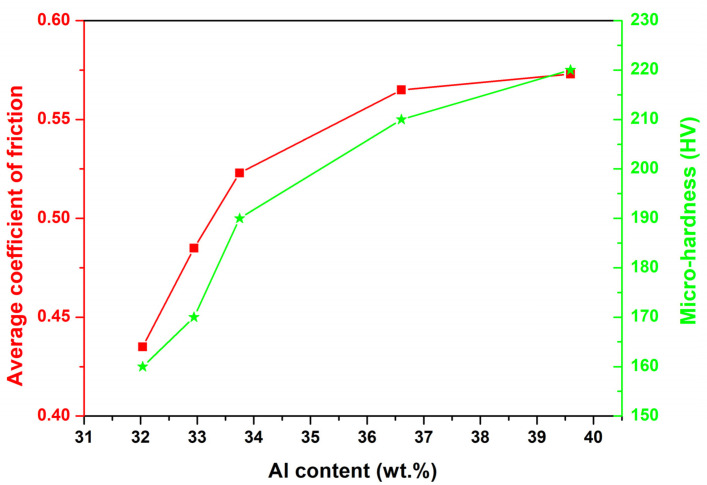
Relationship between Al content and the average coefficient of friction/microhardness.

**Figure 13:**
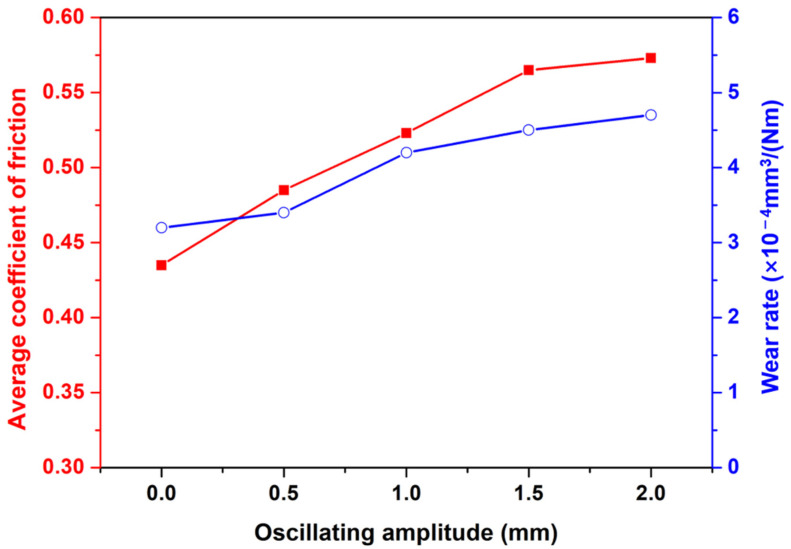
Average coefficient of friction and specific wear rate of samples with different oscillating amplitudes.

The average friction coefficients and wear rates of the samples obtained at different oscillating amplitudes are shown in Figure 11. The average friction coefficients increase with the rise of oscillating amplitude. The tendency of the average friction coefficients remained consistent with the increase in Al content in the top area of the reinforcement, as shown in Figure 12. It was suggested that the increase of the average friction coefficients was mainly attributed to the increase in Al content. The increase in Al content leads to the formation of the more brittle Mg_17_Al_12_ phase, which tends to form a rough friction surface and causes the increase in the average friction coefficients [50]. Concerning the wear rate (Figure 13), the A0 samples exhibit the lowest wear rate of about 3.2 × 10^−4^ mm^3^/(Nm). We noticed that the A0 samples also present the highest hardness. With the increase of the oscillating amplitude, the wear rate increases owing to the decrease in hardness. According to the microstructure and performance analysis, it can be deduced that introducing laser oscillating can suspend the formation of keyhole-induced porosities in the weld seam. With the increase in oscillating amplitude, the distribution of cladded alloy can be improved and can restrain the alloy segregation in the weld seam because of the rapid and fierce stirring by the oscillating laser. However, the hardness and wear resistance of the cladded layer was decreased in the sample with a great oscillating amplitude since the rapid and fierce stirring caused by oscillating laser leads to the diluting of the cladded Al alloy. The diluting of the cladded Al alloy restrains the formation of the brittle Mg_17_Al_12_ phase, and then causes the weakening of hardness and wear resistance of the cladded layer. Comprehensively considering the weld quality (fewer pores) and cladding layer performance (high hardness and wear resistance), oscillating amplitude A = 1.0 might be the optimized selection to clad Al-Mg alloy to improve the wear resistance of the AZ80 alloy.

## 4. Conclusions

Laser–arc hybrid welding with different beam oscillating amplitudes was applied on AZ80 Mg alloy to cladded Al-Mg alloy coating. The laser oscillation amplitude has a considerable influence on the weld surface appearance, profile, elements distribution, and performance.

(1) The penetration depth decreases significantly with the increase in oscillating amplitude, while welds are widened by the increase in the oscillating area of the laser beam.
(2) Alloy segregation and keyhole-induced porosity can be suppressed by the laser beam oscillation because the stirring effect of the laser beam oscillation can promote the diffusion and liquid melt flow.
(3) With the increase in oscillating amplitude, the content of Al in the top area of the seam can be diluted by the laser beam stirring. The diluting of the cladded Al alloy restrains the formation of the brittle Mg_17_Al_12_ phase, and then causes the weakening of hardness and wear resistance of the cladded layer.
(4) The optimized oscillating amplitude was 1 mm, which can produce a weld seam with good appearance, fewer segregation and porosity defects, and acceptable hardness (180 HV) and wear resistance (4.2 × 10^−4^ mm^3^/(Nm)).

## Figures and Tables

**Table 1:** Chemical composition of the base metal and filler metal.

Materials	Reference Composition (wt. %)								
5356 welding wire	Si	Fe	Cu	Mn	Mg	Cr	Zn	Ti	Al
	≤0.25	≤0.4	≤0.1	0.05–0.2	4.5–5.5	0.05–0.2	≤0.1	0.06–0.2	Bal.
AZ80A	Al	Zn	Mn	Si	Fe	Cu	Ni	Be	Mg
	7.8–9.2	020–0.80	0.15–0.50	≤0.10	≤0.05	≤0.05	≤0.005	≤0.01	Bal.

**Table 2:** LAHW processing parameters for cladding 5356 alloy on AZ80A.

Parameters	Value
Laser power	2500 W
Welding speed	2 m/min
Filling speed	4 m/min
Defocused distance	0 mm
Arc current	50−150 A
Oscillation pattern	Circular
Beam oscillating amplitude	0−2.0 mm
Beam oscillating frequency	500 Hz
Shielding gas	Pure argon

## Data Availability

There is no supporting date for this article.
